# Evaluation of Menopausal Syndrome Relief and Anti-Obesity Efficacy of the Korean Fermented Food Doenjang: A Randomized, Double-Blind Clinical Trial

**DOI:** 10.3390/nu16081194

**Published:** 2024-04-17

**Authors:** A Lum Han, Myeong Seon Ryu, Hee-Jong Yang, Do-Youn Jeong, Keum Ha Choi

**Affiliations:** 1 Department of Family Medicine, Wonkwang University Hospital, Iksan 54538, Republic of Korea; 2 Microbial Institute for Fermentation Industry, Sunchang 56048, Republic of Korea; rms6223@naver.com (M.S.R.); yo217@naver.com (H.-J.Y.); godfiltss@naver.com (D.-Y.J.); 3 Departments of Pathology, Wonkwang University Hospital, Iksan 54538, Republic of Korea; jdy2534@korea.kr

**Keywords:** Doenjang, menopausal syndrome, obesity, short-chain fatty acids, microbiome

## Abstract

Foods that help improve menopausal syndrome are being studied worldwide. Doenjang is a traditional Korean fermented soybean food with potential health benefits for menopausal women. In this clinical trial using Doenjang, we aimed to compare the effectiveness of traditional Doenjang and commercial Doenjang in menopausal women. Furthermore, we compared whether Doenjang has a better effect if the number of beneficial microbes is higher. The analyses included the following groups: traditional Doenjang containing either a high dose (HDC; *n* = 18) or low dose (LDC; *n* = 18) of beneficial microbes and commercial Doenjang (CD; *n* = 20). The Kupperman index and hematological changes were examined before and after the use of Doenjang pills. The effects of Doenjang on obesity and body composition were studied before and after ingestion. Lastly, the microorganisms and short-chain fatty acid changes in the stool were compared. The Kupperman index decreased after Doenjang consumption in all three groups, with the greatest decrease in the LDC group. Only the groups that took traditional Doenjang pills exhibited reduced LDL cholesterol. No changes in obesity and inflammation-related indicators were observed. The number of Firmicutes, associated with obesity, decreased in the CD group but the numbers of Bacteroidetes increased in the HDC and CD groups. Thus, traditional Doenjang is more effective in alleviating menopausal syndrome than commercial Doenjang. Further research on the anti-obesity effect or changes in microbiomes and short-chain fatty acids in feces is needed.

## 1. Introduction

As many women undergo menopause, the ovarian function steadily diminishes, resulting in hormonal alterations and subsequent physical and psychological changes. Consequently, symptoms such as hot flashes, sweating, insomnia, genitourinary system atrophy, sleep disturbances, and anxiety occur, and the incidence rates of osteoporosis and cardiovascular disease increase. These symptoms are distinct, although they are collectively known as menopausal syndrome [1]. Hormone replacement therapy (HRT), predominantly estrogen and progesterone, is used to treat menopausal symptoms [1,2]. Estrogen is an effective treatment for hot flashes and other symptoms. However, in specific clinical conditions, estrogen use by some women has been undesirable or contraindicated. Other women may obtain symptomatic relief with estrogen replacement but find the negative effects of estrogen, even in small amounts, to be more unpleasant. Despite the clinical benefits of HRT in postmenopausal women, the compliance rate is low, and estrogen replacement alternatives would be therapeutically beneficial in these cases. The alternatives to estrogen replacement have fewer negative effects, such as cardiovascular disease, venous thromboembolism, and breast cancer, and menopausal-symptom-relieving foods or extracts are gaining popularity [2].

Epidemiological studies have suggested an association between soy consumption and decreased vasomotor symptoms (VMS) such as hot flashes [3]. The soy consumption rates in Asian countries such as Japan, Korea, China, Taiwan, and Indonesia was estimated to be 4–9 times higher than that in Western countries such as the United States, and the incidence rates of hot flashes among Asian women are much lower (10–25%) than among women in Western countries (60–90%) [4]. However, the frequency rates of declarations may skew incidence calculations.

Doenjang, a unique Korean soybean paste, is fermented by a variety of microorganisms and has been made for centuries in homes using traditional methods. *Bacillus subtilis* and molds such as *Rhizopus*, *Mucor*, and *Aspergillus oryzae* from rice straw and local environments are used for inoculation [5]. Soy-based foods have the potential to exert hormone-like activity and antioxidant properties. Several compounds and antioxidants found in soybean and foods made from soybean, including isoflavones, saponins, phytic acid, and phytosterols, have been reported to have potential health benefits, including improving discomfort and reducing oxidative damage [6].

The Kupperman index published by Kupperman et al. is widely used as a diagnostic criterion for menopausal syndrome not only around the world but also in Asia [7]. The symptoms of menopausal syndrome are classified into six categories and include vasomotor disorders, urinary tract symptoms, psychoneurological symptoms, motor symptoms, digestive symptoms, and systemic symptoms [8]. The Kupperman index, which consists of 25 questions identifying 6 categories, can be used to identify the severity and characteristics of menopausal syndrome symptoms. Index point totals of 20 or less can be classified as mild, 20 to 40 points can be classified as moderate, and 40 to 60 points can be classified as severe. Scores of 60 or higher are interpreted as indicating a very serious level of menopausal syndrome [8].

Doenjang supplementation was given to 62 postmenopausal women over 40 years of age with a Kupperman index score of ≥15 in this double-blind, randomized clinical trial. In this clinical trial, we first examined the menopausal syndrome alleviation effects of Doenjang with a high or low content of beneficial microbial strains and the effects of commercially available Doenjang, then attempted to assess the differences between the three groups. The anti-obesity and lipid-lowering benefits of the three types of Doenjang were investigated. Finally, the microbial changes in stool samples after Doenjang ingestion were compared and confirmed. A homeostatic model assessment for insulin resistance (HOMA IR), homeostatic model assessment for β cell (HOMA-β), quantitative insulin sensitivity check index (QUICKI) for insulin resistance, and EQ-5D for quality of life were performed.

This study was designed to evaluate the alleviating effect of Doenjang on menopausal syndrome and to compare whether the effect of traditional Doenjang is better than commercial Doenjang. Furthermore, we compared whether Doenjang had a better effect if the number of beneficial microbes was greater. To date, there has been no clinical investigation analyzing the efficacy of the Doenjang production method in alleviating menopausal syndrome symptoms. This study is valuable as it not only compared the manufacturing methods but also evaluated changes in the microbiome and short-chain fatty acids in feces after consuming Doenjang.

## 2. Materials and Methods

### 2.1. Study Design

This was an eight-week randomized, double-blind clinical trial (registration number KCT 0008958). The participants visited the research center three times. After 4 weeks of Doenjang supplementation, the participants visited the research center to have their vital signs, side effects, and medication compliance evaluated. On the first and last days, markers of menopausal syndrome relief, such as the Kupperman index, were assessed. At the same time, efficacy indicators, including their weight, bioelectrical impedance analysis (BIA) results, inflammatory markers, lipid profiles, and serum blood markers, were evaluated. The participants received the following Doenjang tablets: tablets made from high useful microorganism content (HDC) traditional Doenjang, tablets made from low useful microorganism content (LDC) traditional Doenjang, and tablets made from commercially available Doenjang (CD). Through a microbiome analysis, among the raw materials verified by the Korean Ministry of Food and Drug Safety, the product group with a high content of usable microorganisms and reported to be beneficial was selected as HDC and the product group with a low content was selected as LDC [7]. Ten percent of the 62 study participants who were enrolled dropped out. As a result, 56 participants completed the study.

Screening numbers were given to those who supplied written informed consent to participate in the study. The numbers represented the order of the study participants and ranged from 01 to 62. The number assigned to each participant was used as the participant identification code. All participants were instructed not to take drugs or healthy functional foods other than Doenjang pills and were urged to maintain their usual diet and activity levels.

### 2.2. Participants

Adult women between the ages of 45 and 70 who had reached menopause, were diagnosed with menopausal syndrome, and had a Kupperman index of 15 or above were included in the study. The participants were limited to those who had passed 12 months since their last menstrual period or were diagnosed as menopausal by an obstetrician or gynecologist. Sixty-two volunteers with a body mass index (BMI) ≥ 23 kg/m^2^ were recruited and randomly assigned to three groups. Patients who had undergone HRT within 6 weeks or surgical menopause, those who were contraindicated for hormone therapy, those with acute and severe cardiovascular diseases (heart failure, myocardial infarction, and stroke), those with serious liver function abnormalities, those with a history of clinically significant hypersensitivity to drugs and functional foods, those with a history of psychiatric treatment within 2 months prior to screening, and those with a history of drug or alcohol abuse were excluded. All 62 volunteers provided informed consent prior to treatment. The study protocol was approved by the Institutional Review Board of Wonkwang University Hospital (approval number 2023-04-031).

The participants were continuously instructed not to change their eating patterns and activity levels before and during the clinical trial. The participants completed a physical activity questionnaire based on the Global Physical Activity Questionnaire during their visit [9]. An investigator conducted the interviews based on the KHNANES guidelines [10]. On the visit days, dietary data were collected, and the meals and drinks consumed by subjects during the preceding 24 h were recorded. The participants ingested 6 g of Doenjang tablets (6 g of fermented soybeans) twice a day.

### 2.3. Safety Assessment

Blood chemistry and hematological tests were performed on the participants as part of the screening process. Their white and red blood cell, hemoglobin, hematocrit, platelet counts, total protein, albumin, alanine aminotransferase (ALT), aspartate aminotransferase (AST), blood urea nitrogen (BUN), and creatinine levels were all assessed. The participants’ pulse and blood pressure readings were measured after a 10 min break at each visit. They reported any symptoms that occurred while taking the Doenjang pills.

### 2.4. Indicators of Obesity Assessment

Prior to blood collection, the participants fasted for >12 h. Their total cholesterol (TC), low-density lipoprotein cholesterol (LDL-C), high-density lipoprotein cholesterol (HDL-C), ALT, AST, gamma-glutamyl transferase (GGT), BUN, creatinine, glucose, insulin, and high-sensitivity C-reactive protein levels were analyzed using a Hitachi 7600 automatic analyzer (Hitachi, Tokyo, Japan). Their HOMA IR, HOMA-β, and QUICKI (quantitative insulin sensitivity check index) results were calculated.

### 2.5. Inflammation Marker Assessment

The serum inflammatory marker haptoglobin was measured to determine whether the patients’ inflammation improved after taking Doenjang pills. The blood was allowed to clot for 30 min after collection and then centrifuged at 3000 rpm for 10 min. The separated supernatant was transferred to a microtube, stored at −70 °C, and later analyzed for haptoglobin by a commissioned institution (SCL Healthcare Co., Ltd.; 13 Heungdeok 1-ro, Giheung-gu, Yongin-si, Gyeonggi-do, Republic of Korea).

### 2.6. Assessment of Changes in the Gut Microbiome 

The participants underwent a stool test at the initial visit and another 8 weeks later to examine changes in the intestinal microbiota. Using the MICROBE and ME Stool Collection Kit (Macrogen, Seoul, Republic of Korea), the participants collected 1 g or more of feces, which was then frozen.

### 2.7. Experimental Doenjang Pill Preparation

**Figure 1:**
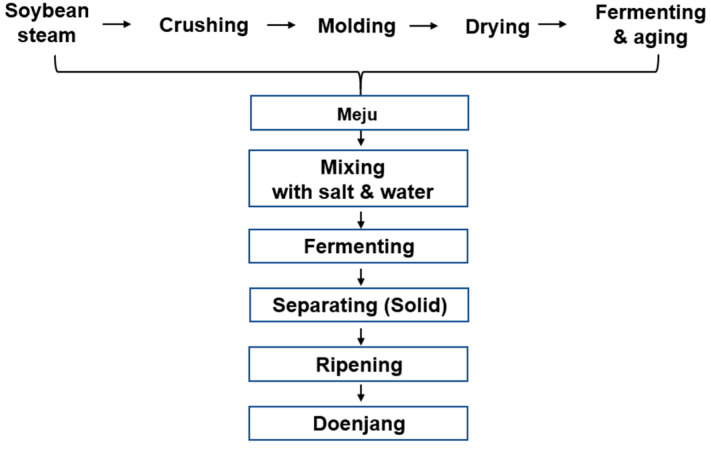
Manufacturing process of traditional Doenjang.

The initial step in making Doenjang is to boil the beans, followed by a 3 day drying process. Subsequently, the processed soybeans go through a fermentation process to become Meju. A saline solution, prepared with sea salt and adjusted to 20–26% salinity, is utilized for fermenting soybean lumps. The fermented soybean lump-to-brine ratio is kept constant at 1:4. Commercial Doenjang is fermented with *Aspergillus oryzae*, whereas traditional Doenjang is fermented with fungi such as *Aspergillus oryzae* and beneficial microorganisms such as *Bacillus subtilis*. Figure 1 depicts the Doenjang production process. The Doenjang was freeze-dried and crushed to create a tablet form for the clinical trials. Before manufacture, it was combined with an excipient in the proportions given in Table 1.

### 2.8. Statistical Analysis

All statistical analyses were performed using PASW Statistics 23 (previously SPSS statistics) (SPSS version 23.0; IMP Corp., Armonk, NY, USA). All data are expressed as the means ± standard error or percentages (%) for categorical variables. Statistical significance was set at *p* < 0.05.

The sample size was chosen to achieve 80% statistical power with a 0.05 alpha. The sample size for each group was calculated using a 20% dropout rate. A chi-square test and an ANOVA were used to detect baseline differences in the frequencies of classified variables between groups. Student’s paired *t*-tests were performed to compare the groups before and after the 8 week intervention period. An expert analyzed the 24 h dietary intake data using Can-Pro 3.0 software (Korean Nutrition Society, Seoul, Republic of Korea).

## 3. Results

### 3.1. Participants

**Figure 2:**
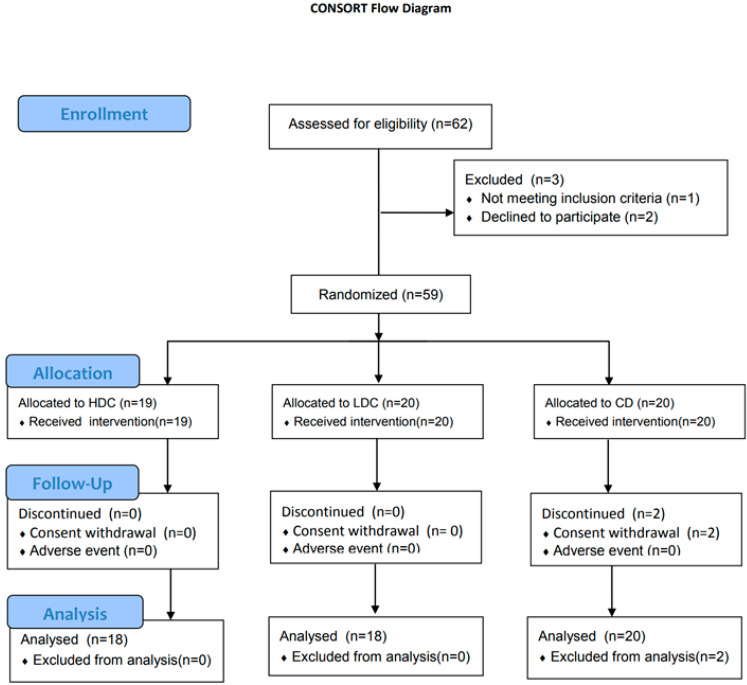
CONSORT flow diagram.

The CONSORT Flow Diagram is shown in Figure 2. A total of 56 participants from the HDC (*n* = 18), LDC (*n* = 18), and CD (*n* = 20) groups were included in the final analysis. None of the participants indicated any adverse events after completing the study.

### 3.2. Anthropometric Parameters

Table 2 shows the overall characteristics of the participants. A cross-analysis was performed for drinking and smoking, as well as a one-way analysis of variance for age, weight, and BMI. In terms of age, weight, height, drinking, smoking, initial weight, and initial BMI, there were no significant differences between the three groups. The dietary intake surveys revealed no significant changes in caloric intake within or between the groups. According to the physical activity survey, there were no significant differences in the metabolic equivalents of the tasks either among or between the groups. The age at menarche, age at menopause, menopause period, and number of births were examined in all subjects, and there were no significant differences between the three groups (Table 3).

### 3.3. Safety Assessment

The blood tests for liver inflammation levels and kidney function, as well as general blood tests, showed no increase after using Doenjang pills. The BUN, uric acid, and T-protein levels in the HDC group decreased after Doenjang ingestion (Table 4). There were significant decreases in BUN, uric acid, and T-protein levels, although this did not affect the safety. No adverse symptoms were noted in any of the groups after taking the Doenjang pills.

### 3.4. Effects on Obesity and Inflammation

The Doenjang showed no anti-obesity effects. In contrast, after the administration of Doenjang pills, the ratio of abdominal fat increased in the HDC and LDC groups. However, the LDL-C levels in the HDC and LDC groups decreased after administering the Doenjang pills. The glucose, insulin, HOMA-IR, HOMA-β, and QUICKI levels were not affected. In all groups, the serum inflammatory marker haptoglobin also showed no changes. There was no change in EQ-5D in any of the three groups after taking Doenjang (Table 5).

### 3.5. Efficacy Evaluation of Kupperman Index Values between the Three Groups

**Figure 3:**
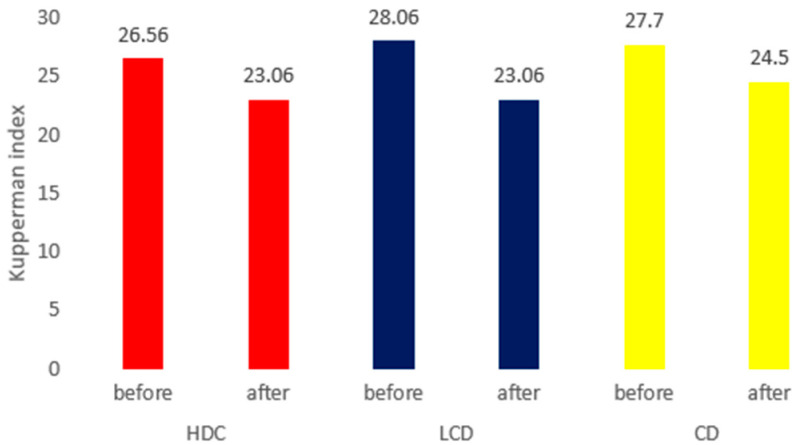
Efficacy evaluation of the Kupperman index values between the three groups. Note: HDC, traditional Doenjang containing a high dose of beneficial microbes; LDC, traditional Doenjang, containing a low dose of effective microbes; CD, commercially prepared Doenjang.

Following the Doenjang administration, the Kupperman index values improved and the total scores significantly decreased in all three groups. In addition to the total score, the change in score for each item was analyzed (Figure 3). The levels of nervousness decreased in all three groups, as did melancholia in the LDC and CD groups, vertigo in the HDC group, and fatigue in the CD group, as well as headache, palpitations, formication, and vaginal dryness in the HDC and LDC groups (Table 6).

### 3.6. Microbiome and Short-Chain Fatty Acid Analysis in Feces

**Figure 4:**
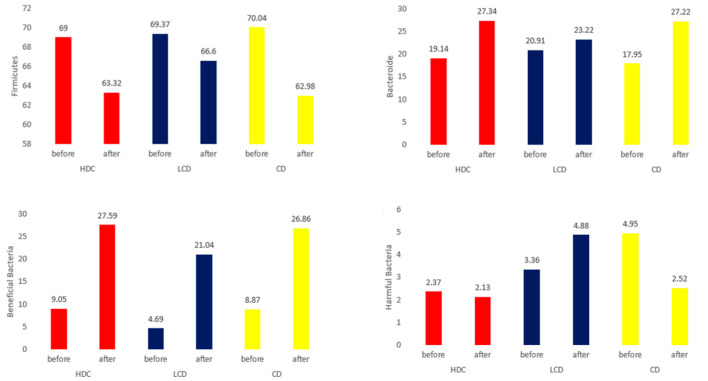
Microbiome analysis of feces. Note: HDC, traditional Doenjang containing a high dose of beneficial microbes; LDC, traditional Doenjang, containing a low dose of effective microbes; CD, commercially prepared Doenjang.

The numbers of Firmicutes decreased in all three groups, although this was statistically significant in only the CD group after Doenjang administration. The numbers of Bacteroidetes increased in all three groups and was statistically significant in only the HDC and CD groups. The Firmicutes-to-Bacteroidetes (F/B) ratio did not decrease in any of the three groups. The presence of beneficial, harmful, and other microorganisms in the stool samples following Doenjang pill ingestion was assessed in all three groups (Figure 4). The numbers of beneficial bacteria increased in all three groups after supplementation, whereas the number of harmful bacteria decreased in only the CD group. The beneficial microorganisms included *Lactobacillus* spp., *Bifidobacterium* spp., *Lactococcus lactis*, *Enterococcus faecium*, and *Bacteroides* spp. Harmful bacteria included *Clostridium perfringens*, *Bacteroides eggerthii*, *Sutterella stercoricanis*, *Ruminococcus torques*, *Parabacteroides merdae*, and *Parabacteroides distasonis* (Table 7). The total short-chain fatty acids increased in the HDC group, although this was not statistically significant, while they decreased in the CD group (Table 8).

## 4. Discussion

This study aimed to compare whether traditional Doenjang is superior to commercial Doenjang in alleviating menopausal syndrome. Furthermore, we compared whether Doenjang would have better effects if it had more beneficial microbes. Based on the manufacturing process, Doenjang is categorized into traditional and commercial forms. Commercial Doenjang is primarily fermented using Koji; consequently, the maturation period is short and the taste is strong, whereas traditional Doenjang contains fungi such as *Aspergillus oryzae* and beneficial microorganisms such as *Bacillus subtilis*, which are involved in the Meju fermentation process. Traditionally manufactured Doenjang is classified into having a high or low effective microbiome content. The group of participants that consumed each Doenjang form was further divided into three groups, and the difference in effect between each group was validated for each group. Changes in obesity-related indicators, lipid profiles, inflammatory indicators, and insulin resistance indicators were confirmed following Doenjang ingestion.

The results of this study showed a statistically significant decrease in the mean Kupperman index following Doenjang consumption, with significant differences between the three groups. The total Kupperman index score decreased the most in the LDC group. The degree of decrease for each item varied between the three groups, and there were no items for which the scores increased. All three groups responded well in the vaginal dryness category, which was investigated separately and was not part of the Kupperman index.

Doenjang did not show anti-obesity, anti-inflammatory, or insulin resistance improvement effects but was effective in reducing the LDL cholesterol levels in the HDC and LDC groups. After supplementation, the numbers of beneficial bacteria increased in all three groups, whereas the number of harmful bacteria decreased in only the CD group.

Menopause is a biological process that can cause various symptoms, including hot flashes and emotional changes, which many women experience. Before 2000, hormonal treatment (HT) was widely used to treat menopausal symptoms such as hot flashes and vaginal dryness [11]. In the late 1990s, HT was used to protect postmenopausal women from chronic conditions such as coronary heart disease (CHD). The Women’s Health Initiative (WHI) conducted a large clinical trial to determine the effectiveness and side effects of HT. As a result of the clinical trial, there were reports of side effects associated with HT, particularly an increased risk of breast cancer in those receiving estrogen and progestin combination therapy [12] and an increased risk of stroke in those receiving estrogen-only therapy [13]. Considering such risks, the use of HT for the primary prevention of menopause-related chronic diseases in women is not recommended. As the findings became widely known, there was a sudden and sustained decline in HT use worldwide [14]. After the WHI trial results were reevaluated the following year, the validity of the initial conclusions was questioned. The revised finding stated that HT was associated with a lower absolute risk of adverse events, including all-cause mortality, in women aged 50–59 years at the time of treatment initiation [15,16]. However, no beneficial effects were observed in women aged 60–69 or 70–79 years at the start of treatment [16]. Despite these encouraging outcomes, many women are reluctant to undergo HT. In addition, after receiving HT, women often complain of minor side effects, although there are no serious side effects [10]. This trend has resulted in many women overcoming menopausal syndrome using various foods or functional products.

Doenjang contains antioxidants such as tocopherol, isoflavones, and phenolic acids derived from soybeans. The isoflavones present in Doenjang include daidzin, genistin, and glycitin-6-o-glucoside [5,6]. Isoflavones have an estrogen-like effect and help to alleviate menopausal symptoms. There are two types of estrogen receptors. ERα is the predominant form in the breast and uterus, whereas ERβ is predominant in the cardiovascular, genitourinary, and skeletal systems. Isoflavones bind weakly to ERα but strongly to Erβ and can be used to relieve menopausal symptoms through this mechanism of action. The basic materials for producing isoflavones include fermented soybean products [17,18].

Natural or surgical menopause results in higher circulating total cholesterol, low-density lipoprotein cholesterol, and triglyceride levels but lower high-density lipoprotein levels, all of which can be reversed by estrogen treatment. Some studies have found phytoestrogens to have similar effects on lipids, whereas others have found no such effects [19].

One study reported that taking an herbal supplement containing isoflavones derived from soybean and red clover for six months reduced the Kupperman index [20]. Another small prospective trial of 51 women who took 60 mg of isoflavones daily for 12 weeks to treat menopausal symptoms found that hot flashes and night sweats were reduced by 57% and 43%, respectively. This treatment had no effect on the levels of estradiol or follicle-stimulating hormone in the blood [18]. As improvements in menopausal symptoms are subjectively assessed, there may be a placebo effect. According to another study, hot flashes were reduced in all patients who consumed isoflavone-rich soy, isoflavone-deficient soy, or whey proteins. However, a similar study found no change in menopausal symptoms after 12 weeks of isoflavone or placebo administration [21]. Nevertheless, recent studies have discouraged using isoflavones to treat menopausal symptoms such as hot flashes [22].

A meta-analysis was conducted on the effects of isoflavone-containing soybean extracts on dyslipidemia. The study’s findings showed statistically significant decreases in LDL cholesterol, total cholesterol, and triglyceride concentrations, as well as a statistically significant increase in HDL cholesterol concentration [23]. A 12 week clinical trial involving 60 overweight Koreans also investigated the anti-obesity and antioxidant effects of Doenjang supplementation. The visceral fat area was significantly reduced in the Doenjang group compared to that of the placebo group, whereas the plasma-free fatty acid and HOMA-IR levels were significantly increased. Although these obesity-related indicators did not change in our study, Doenjang supplementation led to significantly reduced LDL levels [24].

In animal studies, it has been shown that the composition of the intestinal microflora of mice that consumed Doenjang improved significantly. The Doenjang treatment resulted in a significant decrease in Firmicutes but the number of Bacteroidetes increased. These findings have been linked to improved intestinal health by controlling lipopolysaccharide (LPS) levels and inhibiting the production of harmful enzymes [25]. Research has shown that Doenjang significantly stimulates the growth of Bifidobacteria, which in turn inhibits the growth of Enterobacteriaceae, including *Escherichia coli*, and the synthesis of LPS [26,27,28].

When consumed, fermented soybean products, such as Doenjang, boost health by changing the composition of intestinal microorganisms, promoting better metabolism and anti-inflammatory effects [29,30]. The beneficial bacteria in the feces increased following Doenjang consumption in all three groups in this study. There were significantly fewer harmful bacteria in the LDC and CD groups.

Firmicutes and Bacteroidetes, the two most important bacterial phyla in the gastrointestinal tract, have long been known to play important roles in maintaining normal intestinal homeostasis [28,30,31]. The Firmicutes-to-Bacteroidetes (F/B) ratio usually increases, whereas a decrease in the F/B ratio is observed in obesity and inflammatory bowel disease (IBD). Probiotics, as live microorganisms, have beneficial effects on the F/B ratio and obesity when administered in appropriate amounts. Their association with IBD suggests that they have anti-inflammatory properties. Fermented foods, in addition to probiotics, can also improve the F/B ratio [32,33].

Consuming all fermented foods, not just fermented soy products, can affect gut microbiota and their metabolites, especially short-chain fatty acids (SCFAs). SCFAs have several health benefits; they help increase energy expenditure, improve insulin resistance, and reduce the risk of diabetes [34,35]. Acetate is the most abundant SCFA in the colon and it influences cholesterol metabolism. Propionate, which acts as a gluconeogen, inhibits cholesterol synthesis. As a result, conditions that reduce the acetate-to-propionate ratio may lead to improved serum lipid and lower cardiovascular disease risk levels [36]. Propionate and butyrate have been shown to stimulate insulin secretion much more strongly than glucose. This evidence suggests that SCFA production has important effects on host energy metabolism. Although there is still no consensus on the mechanisms and roles of SCFAs in energy homeostasis, several studies have suggested that changes in SCFA levels may affect energy metabolism. Studies have provided evidence to use SCFAs as a possible treatment for diseases such as metabolic syndrome, obesity, and type 2 diabetes [37,38,39,40]. The total short-chain fatty acids increased in the HDC group, although this was not statistically significant, while they decreased in the CD group.

However, this study had several limitations. First, despite our instructions not to change the participants’ eating habits, food choices, activity levels, or exercise routines, we were unable to properly control these variables. Second, because of the short study period, the Doenjang pills could not be supplied for an extended period. Third, because the sample size was limited, the results cannot be generalized. As this study was intended to verify the superiority of traditional Doenjang, a control group was not set up. Therefore, there may be bias caused by a placebo effect.

## 5. Conclusions

This study aimed to not only compare the effect of Doenjang in relieving menopausal symptoms but also study whether traditional Doenjang is better than commercial Doenjang. Furthermore, we compared whether Doenjang would have better effects if it had more beneficial microbes. The Kupperman index scores decreased after Doenjang consumption in all three groups, with the greatest decrease in the LDC group, indicating that traditional Doenjang may be a better alternative for alleviating menopausal syndrome in postmenopausal women. The LDL cholesterol reduction provided further protection against cardiovascular disease. The reduction in LDL cholesterol was found exclusively when using traditional Doenjang, demonstrating its superiority. An increase in beneficial bacteria in the feces, a hallmark of fermented foods, can be expected to improve the intestinal environment. Although the traditional Doenjang did not outperform the commercial Doenjang in alleviating menopausal symptoms, better results are expected in the future with increased supplementation.

## Figures and Tables

**Table 1:** Composition of Doenjang pills.

Ingredient	HDC		LDC		CD	
	Content (g)	Ratio (%)	Content (g)	Ratio (%)	Content (g)	Ratio (%)
Freeze-dried Doenjang powder	6	100	6	100	6	100
Total	6	100	6	100	6	100

Note: HDC, traditional Doenjang containing a high dose of beneficial microbes; LDC, traditional Doenjang containing a low dose of effective microbes; CD, commercially prepared Doenjang.

**Table 2:** General characteristics of the participants.

Value	Group			
	HDC (*n* = 18)	LDC (*n* = 18)	CD (*n* = 20)	*p*-Value
Drinking (*n*)	18 (100)	16 (88.9)	20 (100)	0.112
Smoking (*n*)	18 (100)	17 (94.4)	20 (100)	0.341
Age	59.61 ± 5.12	59.56 ± 5.17	60.35 ± 4.08	0.848
Weight	63.68 ± 6.99	65.08 ± 7.82	64.19 ± 6.26	0.833
BMI	26.29 ± 2.49	26.24 ± 3.13	26.57 ± 3.07	0.935

Note: HDC, traditional Doenjang containing a high dose of beneficial microbes; LDC, traditional Doenjang containing a low dose of effective microbes; CD, commercially prepared Doenjang; BMI, body mass index. Values are presented as the means ± standard deviation or number (percentage).

**Table 3:** Menopause-related factors.

Value	Group			
	HDC (*n* = 18)	LDC (*n* = 18)	CD (*n* = 20)	*p*-Value
Menarche age	15.22 ± 1.31	14.78 ± 1.35	15.8 ± 1.24	0.060
Menopause age	50.94 ± 4.12	52.11 ± 2.42	52.6 ± 2.23	0.237
Menopause period	105.11 ± 80.96	88.67 ± 61.38	93.75 ± 57.55	0.753
Number of births	2.28 ± 0.67	1.89 ± 0.9	2.1 ± 0.72	0.321

Note: HDC, traditional Doenjang containing a high dose of beneficial microbes; LDC, traditional Doenjang, containing a low dose of effective microbes; CD, commercially prepared Doenjang. Values are presented as the means ± standard deviation or number (percentage).

**Table 4:** Hematological safety measurements.

Value	Group								
	HDC (*n* = 18)			LDC (*n* = 18)			CD (*n* = 20)		
	Before	After	*p*-Value	Before	After	*p*-Value	Before	After	*p*-Value
WBC	5.89 ± 1.53	5.79 ± 1.25	0.647	5.94 ± 1.5	6.1 ± 1.62	0.398	6.41 ± 1.65	6.46 ± 1.93	0.887
RBC	4.42 ± 0.39	4.35 ± 0.37	0.165	4.26 ± 0.27	4.25 ± 0.24	0.833	4.41 ± 0.28	4.41 ± 0.29	1.000
Hemoglobin	13.49 ± 0.87	13.2 ± 0.86	0.084	13.09 ± 0.91	12.91 ± 0.86	0.312	13.59 ± 0.74	13.48 ± 0.64	0.376
Hematocrit	39.55 ± 2.51	38.78 ± 2.41	0.111	38.39 ± 2.69	38.27 ± 2.24	0.789	39.84 ± 2.17	39.72 ± 1.66	0.803
GGT	24.89 ± 10.78	25.06 ± 20.36	0.956	34.72 ± 26.45	32.11 ± 18.96	0.504	24.8 ± 17.35	22.9 ± 12.13	0.311
AST	26.22 ± 7.45	26.22 ± 9.93	1.000	24.67 ± 6.43	25.78 ± 10.54	0.559	28.15 ± 10.44	27.5 ± 10.22	0.778
ALT	25.22 ± 13.22	25.61 ± 18.36	0.819	23.56 ± 12.97	24.17 ± 15.14	0.834	25.15 ± 16.44	25.45 ± 19.28	0.857
BUN	14.96 ± 3.27	13.17 ± 3.04	0.040	15.79 ± 3.23	15.04 ± 4.35	0.507	13.92 ± 3.59	13.06 ± 3.4	0.151
Creatinine	0.68 ± 0.1	0.66 ± 0.11	0.370	0.7 ± 0.1	0.69 ± 0.1	0.794	0.67 ± 0.11	0.67 ± 0.08	0.954
Uric acid	4.89 ± 0.88	4.46 ± 0.79	0.010	4.63 ± 1.13	4.46 ± 1.22	0.157	4.62 ± 1.07	4.25 ± 0.92	0.026
T-protein	7.29 ± 0.32	7.11 ± 0.28	0.004	7.11 ± 0.42	7.01 ± 0.29	0.132	7.17 ± 0.32	7.07 ± 0.32	0.222
Albumin	4.47 ± 0.19	4.4 ± 0.13	0.111	4.52 ± 0.23	4.44 ± 0.19	0.084	4.5 ± 0.18	4.47 ± 0.18	0.390
T-bilirubin	0.73 ± 0.29	0.74 ± 0.24	0.879	0.76 ± 0.21	0.72 ± 0.22	0.269	0.78 ± 0.29	0.86 ± 0.42	0.206
LD	172.56 ± 45.78	179.67 ± 35.85	0.513	193.94 ± 23.05	196.06 ± 27.97	0.577	211.35 ± 40.1	211.05 ± 49.83	0.977
ALP	74.78 ± 22.4	74.61 ± 20.11	0.939	82.72 ± 30.8	82.11 ± 25.72	0.800	79.4 ± 15.4	78.55 ± 14.51	0.763
CK	103.17 ± 51.57	109.33 ± 70.14	0.738	130.33 ± 82.46	131.11 ± 88.02	0.934	172.45 ± 239.07	130.8 ± 54.94	0.384

Note: WBC, white blood cell; RBC, red blood cell; GGT, gamma-glutamyl transferase; ALT, alanine aminotransferase; AST, aspartate aminotransferase; BUN, blood urea nitrogen; T-protein, total protein; T-bilirubin, total bilirubin; LD, lactate dehydrogenase; ALP, alkaline phosphatase; CK, creatine kinase.

**Table 5:** Efficacy evaluation between the three groups.

Value	Group								
	HDC (*n* = 18)			LDC (*n* = 18)			CD (*n* = 20)		
	Before	After	*p*-Value	Before	After	*p*-Value	Before	After	*p*-Value
SBP	130.56 ± 9.68	132.5 ± 13.04	0.545	137 ± 12.17	136.33 ± 11.81	0.783	133.55 ± 10.87	132.85 ± 13.57	0.794
DBP	77.06 ± 8.15	76.78 ± 9.02	0.921	80 ± 7.98	77.33 ± 7.2	0.098	76 ± 8.72	78.6 ± 7.13	0.249
Pulse	74.17 ± 9.97	78.28 ± 9.7	0.177	76 ± 8.13	74.06 ± 10.94	0.447	77.2 ± 9.07	78.5 ± 10.52	0.454
WC	87.83 ± 4.09	87.54 ± 4.34	0.362	91.35 ± 6.35	90.71 ± 6.82	0.282	90.52 ± 5.89	90.41 ± 5.82	0.089
HC	92.4 ± 16.57	95.92 ± 5.79	0.303	97.12 ± 4.42	97.12 ± 4.35	1.000	98.98 ± 5.75	99 ± 5.78	0.894
WHR	0.92 ± 0.04	0.92 ± 0.04	0.772	0.94 ± 0.04	0.93 ± 0.04	0.381	0.92 ± 0.04	0.91 ± 0.04	0.021
Weight	63.68 ± 6.99	63.89 ± 7.25	0.303	65.08 ± 7.82	65.06 ± 7.49	0.933	64.19 ± 6.26	63.91 ± 6.29	0.229
BMI	26.29 ± 2.49	26.36 ± 2.58	0.426	26.24 ± 3.13	26.25 ± 3.1	0.942	26.57 ± 3.07	26.46 ± 3.06	0.267
BFM	24.25 ± 5.21	24.43 ± 5.29	0.624	23.49 ± 4.41	23.84 ± 4.46	0.065	24.02 ± 5.54	24.06 ± 5.36	0.838
PBF	37.79 ± 4.15	37.87 ± 3.98	0.887	35.96 ± 3.71	36.51 ± 3.64	0.0501	37.15 ± 6.1	37.36 ± 5.67	0.444
FFM	39.43 ± 2.96	39.46 ± 2.71	0.937	41.59 ± 4.91	41.22 ± 4.45	0.126	40.18 ± 3.82	39.87 ± 3.54	0.156
AFR, Abdominal fat rate	0.91 ± 0.04	0.93 ± 0.05	0.020	0.90 ± 0.05	0.93 ± 0.06	<0.0001	0.91 ± 0.06	0.93 ± 0.06	0.150
BMR	1221.56 ± 64.14	1222.44 ± 58.32	0.907	1268.39 ± 106.16	1260.33 ± 95.94	0.124	1237.55 ± 82.39	1230.95 ± 76.12	0.168
hs-CRP	1.51 ± 2.02	1.27 ± 1.34	0.631	1.12 ± 1.11	1.33 ± 1.76	0.645	1.19 ± 1.34	1.05 ± 1.15	0.599
ESR	8.5 ± 6.53	9.39 ± 5.76	0.495	6.39 ± 5.29	8.28 ± 9.56	0.314	5.45 ± 4.14	5.85 ± 3.12	0.681
Haptoglobin	86.28 ± 38.8	86.94 ± 39.14	0.917	106.72 ± 40.81	111.94 ± 55.76	0.472	97.8 ± 48.89	99.65 ± 43.98	0.809
HDL	56.06 ± 12.3	54.06 ± 14.19	0.285	52.22 ± 7.76	58.72 ± 20.43	0.141	53.9 ± 10.35	54.7 ± 9.52	0.583
LDL	134.28 ± 47.29	116.89 ± 42.05	0.015	123.06 ± 43.1	109 ± 38.23	0.015	117.15 ± 30.17	114.3 ± 32.12	0.606
TC	215.89 ± 48.07	199.28 ± 43.18	0.037	203.78 ± 49.23	196.33 ± 41.73	0.117	191.8 ± 34.91	192.8 ± 35.15	0.860
Glucose	107.06 ± 9.73	106.72 ± 8.99	0.823	113.72 ± 22.14	111.5 ± 24.81	0.379	117.95 ± 28.83	121.95 ± 39.13	0.290
Insulin	6.85 ± 4.28	6.87 ± 3.55	0.989	6.97 ± 4.1	8.1 ± 4.88	0.087	8.23 ± 6.17	7.86 ± 5.04	0.564
HOMA IR	33.32 ± 22.52	32.82 ± 17.62	0.901	37.97 ± 31.13	44.29 ± 37.67	0.191	42.72 ± 34.35	42.16 ± 27.21	0.900
HOMA β	1.31 ± 0.76	1.33 ± 0.67	0.879	1.22 ± 0.54	1.42 ± 0.64	0.055	1.48 ± 1.03	1.38 ± 0.9	0.300
QUICKI	0.36 ± 0.04	0.36 ± 0.03	0.367	0.36 ± 0.04	0.36 ± 0.05	0.675	0.35 ± 0.04	0.35 ± 0.05	0.808
EQ-5D	0.92 ± 0.10	0.93 ± 0.04	0.612	0.90 ± 0.07	0.92 ± 0.06	0.095	0.92 ± 0.06	0.93 ± 0.04	0.909

Note: HDC, traditional Doenjang containing a high dose of beneficial microbes; LDC, traditional Doenjang, containing a low dose of effective microbes; CD, commercially prepared Doenjang; SBP, systolic blood pressure; DBP, diastolic blood pressure; HC, hip circumference; WC, waist circumference; WHR, waist–hip ratio; BMI, body mass index; BFM, body fat mass; PBF, percentage body fat; FFM, fat free mass; AFR, abdominal fat rate; BMR, basal metabolic rate; hs-CRP, high-sensitivity C-reactive, ESR, erythrocyte sedimentation rate; HDL, high-density cholesterol; LDL, low-density cholesterol; TC, total cholesterol; HOMA IR, homeostatic model assessment for insulin resistance; HOMA-β, homeostatic model assessment for β cells; QUICKI, quantitative insulin sensitivity check index; EQ-5D, EuroQol-5D.

**Table 6:** Efficacy evaluation of the Kupperman index values between the three groups.

Value	Group								
	HDC (*n* = 18)			LDC (*n* = 18)			CD (*n* = 20)		
	Before	After	*p*-Value	Before	After	*p*-Value	Before	After	*p*-Value
Vasomotor	8.44 ± 3.03	8 ± 2.74	0.331	8.67 ± 3.14	7.78 ± 2.56	0.104	9.60 ± 2.72	8.60 ± 2.68	0.056
Paresthesia	3.33 ± 1.94	3.33 ± 1.19	1.000	2.56 ± 1.92	2.44 ± 1.10	0.772	3.20 ± 1.77	3.30 ± 1.17	0.716
Insomnia	3.44 ± 2.45	3.78 ± 1.35	0.507	4.00 ± 1.68	3.67 ± 1.41	0.187	4.00 ± 1.72	4.20 ± 1.44	0.330
Nervousness	2.00 ± 2.06	0.89 ± 1.23	0.028	2.89 ± 2.08	1.83 ± 2.12	0.010	1.80 ± 2.14	0.80 ± 1.20	0.004
Melancholia	0.44 ± 0.70	0.11 ± 0.32	0.055	0.56 ± 0.86	0.17 ± 0.38	0.049	0.55 ± 0.89	0.20 ± 0.70	0.031
Vertigo	1.39 ± 0.70	1.06 ± 0.54	0.029	1.44 ± 0.92	1.22 ± 0.73	0.260	1.35 ± 0.88	1.20 ± 0.62	0.419
Fatigue	2.22 ± 0.88	2.5 ± 0.62	0.135	2.78 ± 0.43	2.72 ± 0.67	0.579	2.30 ± 0.92	2.75 ± 0.44	0.083
Headache	1.22 ± 0.65	0.78 ± 0.65	0.007	1.28 ± 0.83	0.83 ± 0.71	0.007	1.15 ± 0.88	0.95 ± 0.83	0.297
Arthralgia and myalgia	2.28 ± 0.96	2.22 ± 0.73	0.790	2.44 ± 0.78	2.11 ± 0.58	0.111	2.30 ± 0.80	2.30 ± 0.57	1.000
Palpitation	1.11 ± 1.02	0.22 ± 0.55	0.001	1.06 ± 1.00	0.22 ± 0.55	0.001	1.05 ± 1.10	0.20 ± 0.41	0.001
Formication	0.67 ± 1.03	0.17 ± 0.51	0.015	0.39 ± 0.78	0.06 ± 0.24	0.055	0.40 ± 0.68	0 ± 0	0.017
Total	26.56 ± 5.03	23.06 ± 4.09	0.001	28.06 ± 5.60	23.06 ± 5.15	0.000	27.7 ± 4.75	24.5 ± 4.36	0.001
Vaginal dryness	2.11 ± 1.02	1.67 ± 0.84	0.002	2.00 ± 1.06	1.53 ± 0.87	0.007	2.15 ± 0.93	1.6 ± 0.99	0.024

Note: HDC, traditional Doenjang containing a high dose of beneficial microbes; LDC, traditional Doenjang, containing a low dose of effective microbes; CD, commercially prepared Doenjang.

**Table 7:** Microbiome analysis of feces.

Value	HDC			LDC			CD		
	Before	After	*p*-Value	Before	After	*p*-Value	Before	After	*p*-Value
Firmicutes (%)	69.00 ± 13.00	63.32 ± 11.11	0.140	69.37 ± 10.66	66.60 ± 13.98	0.310	70.04 ± 11.51	62.98 ± 16.42	0.001
Bacteroidetes (%)	19.14 ± 9.86	27.34 ± 13.48	0.035	20.91 ± 8.83	23.22 ± 14.28	0.411	17.95 ± 10.33	27.22 ± 16.96	0.001
F/B	6.41 ± 8.74	13.77 ± 46.18	0.529	5.42 ± 7.08	5.20 ± 6.46	0.914	41.88 ± 110.40	19.17 ± 56.62	0.398
Beneficial Bacteria	9.05 ± 7.18	27.59 ± 11.52	<0.0001	4.69 ± 2.88	21.04 ± 6.88	<0.0001	8.87 ± 8.51	26.86 ± 10.20	<0.0001
Harmful Bacteria	2.37 ± 1.58	2.13 ± 1.57	0.658	3.36 ± 4.92	4.88 ± 9.26	0.419	4.95 ± 5.61	2.52 ± 2.17	0.046
Others	88.58 ± 7.07	70.28 ± 12.03	<0.0001	91.94 ± 4.79	74.08 ± 9.50	<0.0001	86.18 ± 11.05	70.62 ± 10.52	<0.0001

Note: HDC, traditional Doenjang containing a high dose of beneficial microbes; LDC, traditional Doenjang, containing a low dose of effective microbes; CD, commercially prepared Doenjang. Values are presented as the means ± standard deviation or number (percentage).

**Table 8:** Short-chain fatty acid analysis of feces.

Value	HDC			LDC			CD		
	Before	After	*p*-Value	Before	After	*p*-Value	Before	After	*p*-Value
Acetic acid	40.32 ± 33.28	56.15 ± 50.44	0.279	48.64 ± 24.71	49.2 ± 26.6	0.953	56.3 ± 38.26	41.72 ± 24.9	0.148
Propionic acid	20.21 ± 19.53	35.9 ± 39.55	0.297	22.84 ± 15.42	22.15 ± 25.87	0.930	34.4 ± 27.44	14.54 ± 10.16	0.020
Butytic acid	89.68 ± 33.31	84.61 ± 30.76	0.631	91.35 ± 18.32	85.89 ± 27.54	0.558	94.43 ± 27.64	74.24 ± 36.75	0.040
Total	143.46 ± 68.85	164.68 ± 105.29	0.490	162.83 ± 48.97	157.23 ± 56.05	0.791	173.71 ± 75.25	124.77 ± 62.63	0.018

Note: HDC, traditional Doenjang containing a high dose of beneficial microbes; LDC, traditional Doenjang, containing a low dose of effective microbes; CD, commercially prepared Doenjang. Values are presented as the means ± standard deviation or number (percentage).

## Data Availability

The original contributions presented in the study are included in the article, further inquiries can be directed to the corresponding author.
